# Immediate Rise in Intraocular Pressure After First-Time Intravitreal Injection of Bevacizumab

**DOI:** 10.7759/cureus.38916

**Published:** 2023-05-11

**Authors:** Pir S Mahar, Sohail Bukhari, Ayisha Shakeel, Abdul Sami Memon, Tauseef Mahmood

**Affiliations:** 1 Ophthalmology, Aga Khan University Hospital, Karachi, PAK; 2 Ophthalmology, Isra Postgraduate Institute of Ophthalmology, Karachi, PAK; 3 Ophthalmology, Fazaia Medical College, Air University, Islamabad, PAK

**Keywords:** anti -vegf, chorioretinal disease, bevacizumab, intravitreal injection, intraocular pressure

## Abstract

Objective

This study aims to report an immediate change in intraocular pressure (IOP) after the first injection of bevacizumab.

Materials and methods

An interventional case series was carried out at Isra Postgraduate Institute of Ophthalmology, Al-Ibrahim Eye Hospital, Karachi, from November 2018 to April 2020. All patients with various chorioretinal diseases requiring anti-VEGF treatment were included in the study. Patients with a history of previous anti-VEGF or steroid injections and personal or family history of glaucoma were excluded. Bevacizumab in a dose of 1.25 mg (0.05 ml) was injected intravitreally under topical anesthesia maintaining sterile aseptic conditions in the operating room. IOP was checked one hour prior to the injection, and hourly monitoring of it was continued for the next six hours. Data were analyzed using SPSS Statistics to compare the mean IOP readings before and after injection.

Results

A total of 191 eyes of 147 patients were included in the study. Among them, 92 (62.58%) were male and 55 (37.41%) were female with a mean age of 45.5 ± 8.8 years. The mean pre-injection IOP was measured as 12.12 ± 2.11 mmHg. The frequency of IOP elevation of ˃ 21 mmHg was observed in 169 (88.5%) eyes at five minutes, 104 (54.5%) eyes at 30 minutes, 33 (17.3%) eyes at one hour, and 16 (8.4%) eyes at two hours. The raised mean post-operative IOP was 30.44 ± 6.53 mmHg at five minutes, 26.27 ± 4.65 mmHg at 30 minutes, 26.12 ± 3.31 mmHg at one hour, and 25.63 ± 3.03 mmHg at two hours. The IOP reduced to pre-injection value at three hours measuring 12.12 ± 2.11 mmHg and continued to stay at that level for the next three hours.

Conclusions

The majority of the eyes receiving first-time intravitreal bevacizumab injection showed a significant increase in IOP level within five minutes to two hours post-injection.

## Introduction

Anti-VEGF agents have been the treatment of choice for many choroidal and retinal vascular diseases. Bevacizumab (Avastin, Genentech) is a humanized monoclonal antibody to vascular endothelial growth factor approved for the treatment of colorectal cancer. It has been used off-label intravitreally since 2005 and is one of the common anti-VEGF agents used extensively due to its low cost and comparative usefulness [1,2]. The widespread use of this agent is not without side effects ranging from benign subconjunctival hemorrhage and corneal abrasion to more serious lens injury and endophthalmitis [3].

The transient and sustained rise in IOP can be expected with intravitreal injections due to an increase in vitreous volume and the toxic effect of the injected drug on the trabecular meshwork [4]. Any significant increase in intraocular pressure (IOP) can have a deteriorating effect on an already compromised optic disc, especially in glaucoma patients.

With the introduction of other anti-VEGF compounds available in the market for the treatment of various chorioretinal disorders, Bakri et al. used triamcinolone, pegaptanib, and bevacizumab intravitreally. They recorded an immediate rise (at 10 minutes) of more than 10mmHg in IOP in 27.6% of eyes receiving bevacizumab, 33.3% of eyes receiving triamcinolone, and 36.2% of eyes receiving pegaptanib [5].

The sustained rise in IOP after repeated intravitreal anti-VEGF agents was first reported by Adelman in 2010 [6]. In his cohort of 116 patients with age-related macular degeneration (ARMD), 34% of patients developed raised IOP measuring up to 31.7 mmHg.

Since then, multiple reports have followed, showing a transient and sustained rise in IOP following anti-VEGF injections [7-9]. However, direct comparison of these studies is difficult due to the different compounds used intravitreally, the different nature of the studies, the different types of tonometers used for monitoring the IOP, and, above all, the time period when IOP measurements were done post-injection.

Various researchers have investigated a variety of risk factors for the potential rise in IOP. This includes multiple injections, the absence of post-injection sub-conjunctival reflux, smaller needles, tunneled injection technique, and previous diagnosis of glaucoma [10]. Bracha et al. suggested that a transient rise in IOP after injecting 50 microliters into an average vitreous volume of 4.5 to 5 mL occurs due to the increase in vitreous volume [11].

To keep the multiple arrays of suggestions, we wanted to find out the change in IOP when an intravitreal injection is given for the first time in non-glaucomatous patients.

We, therefore, conducted this prospective study to monitor the immediate change (up to six hours) in IOP of patients receiving bevacizumab injection for the first time.

## Materials and methods

An interventional study was conducted at the Isra Postgraduate Institute of Ophthalmology, Al-Ibrahim Eye Hospital, Karachi from November 2018 to April 2020. The study was approved by the Ethical Review Committee of the institute and was performed in accordance with the Declaration of Helsinki. The sample size was drawn through OpenEpi online sample calculator by using an expected population of 1500 patients receiving intravitreal injection per year, and the difference between the mean pre-IOP (16.9 mmHg) and post-IOP (33.9 mmHg) after injection was calculated at 17 mmHg as the anticipated frequency, keeping 95% confidence interval and 5% margin of error [12]. The required sample size was found to be 190. All patients signed informed consent forms describing the potential risks and benefits of the procedure. All patients having various retinal and choroidal vascular diseases and receiving their first injection of bevacizumab (Avastin, Genentech USA) were included in this study. We excluded patients with a personal or family history of glaucoma, an IOP of more than 20 mmHg, and a history of receiving any intravitreal anti-VEGF and steroid injection in the past.

All enrolled patients underwent a complete ocular examination including best-corrected visual acuity, slit-lamp biomicroscopic examination of the anterior segment, gonioscopy, and recording of IOP by Goldmann Applanation Tonometer. A dilated fundus examination was carried out with a + 90 Diopter lens (Volk, UK) and an indirect ophthalmoscope. Ancillary tests like optical coherence tomography, optical coherence tomography angiography, and fundus fluorescein angiography were obtained in all patients.

All injections were performed under aseptic conditions in the operating theater under topical anesthesia (Alcaine, Proparacaine, Alcon, Pakistan). A dose of 1.25 mg (0.05 ml) was injected intravitreally 3.5 mm from the limbus using a half-inch-long 29-gauge needle in the superior-temporal quadrant. (This needle has a wall thickness of 0.76 mm, an outer diameter of 0.337 mm, and an inner diameter of 0.184 mm.) After injection, a sterile cotton swab was placed on the injection site to prevent reflux of the given drug. A drop of 5% povidone-iodine was instilled before and after the procedure. All procedures were performed between 10:00 to 11:30 AM.

The IOPs of all patients were checked one hour before receiving intravitreal bevacizumab injection and subsequently at five minutes, 30 minutes, and one, two, three, four, and six hours post-injection. The IOPs were recorded by the same observer throughout the study period.

We injected bevacizumab in both eyes of 44 patients as no cross-over effect of the drug in the other eye causing a rise in IOP is described. The pressure spike was found to be similar in patients whose both eyes were injected.

Data were analyzed using SPSS Statistics version 23.0 (IBM Corp. Released 2015. IBM SPSS Statistics for Windows, Version 23.0. Armonk, NY: IBM Corp.). All continuous variables were presented in Mean±SD, and categorical variables were presented as frequency and percentages. The chi-square test or Fisher’s exact test was used to check the association between two categorical variables. To compare the mean IOP of diagnosis for each time interval, repeated measures ANOVA was used. The baseline and postoperative IOP at each follow-up were analyzed using paired sample t-tests. P-values of ≤ 0.05 were considered statistically significant.

## Results

**Figure 1:**
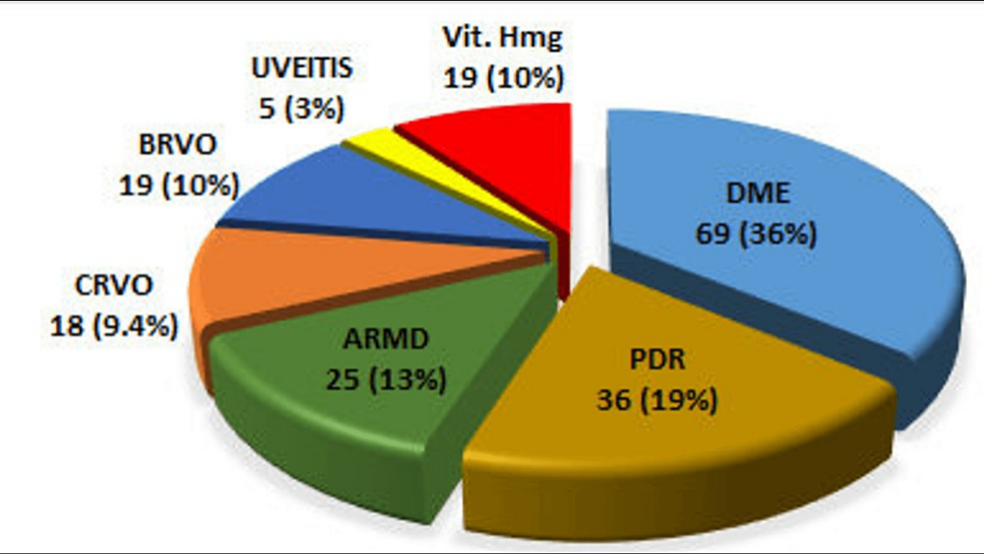
Frequency of eyes with primary diagnosis DME: diabetic macular edema, PDR: proliferative diabetic retinopathy, ARMD: age-related macular degeneration, CRVO: central retinal vein occlusion, BRVO: branch retinal vein occlusion, Vit. Hmg: vitreous hemorrhage

One hundred and ninety-one eyes of 147 patients were included in this study. Out of 147 patients, 92 (62.58%) were male and 55 (37.41%) were female, with a male-to-female ratio of 1.67:1. The mean age of patients was 54.5 ± 8.8 years (range 35- 5 years). The distribution of our patients with primary diagnosis is shown in Figure 1.

**Figure 2:**
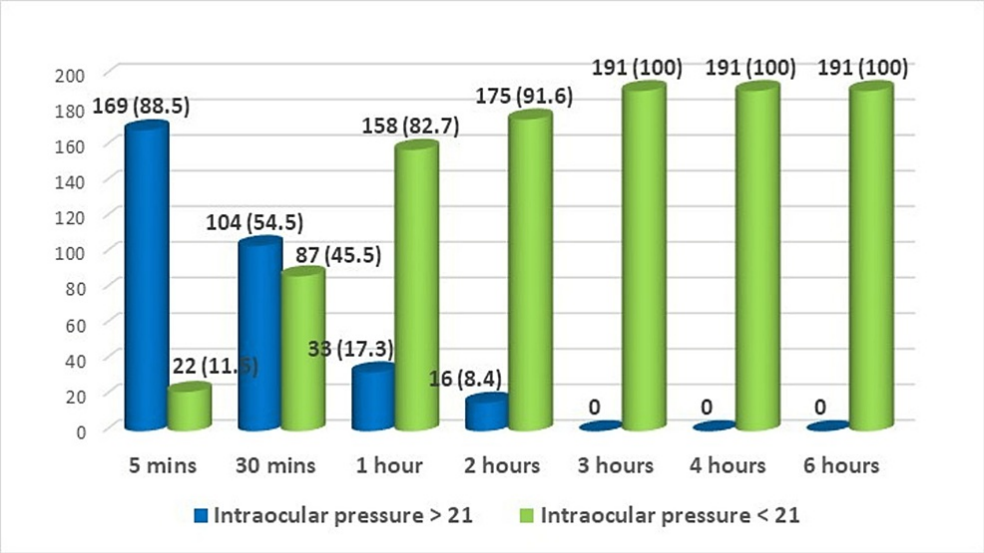
Frequency of raised and normal IOP with time intervals

Out of 191 eyes, 119 (62.3%) were phakic and 72 (37.7%) were pseudophakic. The frequency of IOP elevation >21 mmHg following intravitreal bevacizumab was observed in 169 eyes (88.5%) at five minutes, 104 eyes (54.5%) at 30 minutes, 33 eyes (17.3%) at one hour, and 16 eyes (8.4%) at two hours, with no increase in IOP observed at three, four, and six hours (Figure 2).

The maximum rise in IOP at all time intervals was noticed in patients with diabetic macular edema (DME), followed by proliferative diabetic retinopathy (PDR), whereas the least increase in IOP was seen in patients with posterior uveitis (p < 0.001) (Table 1).

**Table 1:** Correlation of raised IOP (≥ 21 mmHg) with the diagnosis IOP: intraocular pressure, DME: diabetic macular edema, PDR: proliferative diabetic retinopathy, ARMD: age-related macular degeneration, CRVO: central retinal vein occlusion, BRVO: branch retinal vein occlusion, Vit. Hmg: vitreous hemorrhage

Primary diagnosis	Raised IOP at different time intervals (mmHg)				p-value
	Five minute	30 minute	One hour	Two hours	
	≥ 21	≥ 21	≥ 21	≥ 21	0.001
DME	60 (31.4)	32 (16.8)	7 (3.7)	4 (2.1)	
PDR	31 (16.2)	23 (12.0)	6 (3.1)	1 (0.5)	
ARMD	19 (9.9)	8 (4.2)	3 (1.6)	1 (0.5)	
CRVO	18 (9.4)	15 (7.9)	6 (3.1)	2 (1.0)	
BRVO	17 (8.9)	9 (4.7)	1 (0.5)	1 (0.5)	
UVIETIS	5 (2.6)	1 (0.5)	0 (0.0)	0 (0.0)	
Vit. Hmg	19 (9.9)	16 (8.4)	10 (5.2)	7 (3.7)	
Total no. of eyes with IOP ≥ 21	169 (88.5%)	104 (54.5%)	33 (17.3%)	16 (8.4%)	

The mean IOP one hour before injection was recorded at 12.12 ± 2.11 mmHg. It increased to 30.44 ± 6.53 mmHg (p < 0.001) after five minutes, 26.27 ± 4.65 mmHg (p < 0.001) after 30 minutes, 26.12 ± 3.31 mmHg (p < 0.001) after one hour, and 25.63 ± 3.03 mmHg (p < 0.001) after two hours post-injection (Table 2).

**Table 2:** IOP with primary diagnosis at different time intervals IOP: intraocular pressure, DME: diabetic macular edema, PDR: proliferative diabetic retinopathy, ARMD: age-related macular degeneration, CRVO: central retinal vein occlusion, BRVO: branch retinal vein occlusion, Vit. Hmg: vitreous hemorrhage The mean of post-operative IOP at each interval is compared for the same diagnosis.

Primary diagnosis	Frequency (n=191 eyes)	Baseline mean IOP (mm Hg)	Mean IOP at five minutes (mmHg)	Mean IOP at 30 minutes (mmHg)	Mean IOP at one hour (mmHg)	Mean IOP at two hours (mmHg)	Mean IOP at three hours (mmHg)	Mean IOP at four hours (mmHg)	Mean IOP at six hours (mmHg)	p-value
DME	69	11.74	27.41	24.83	23.77	22.70	18.24	14.94	11.24	0.001
PDR	36	12.61	28.94	22.78	24.17	23.40	16.19	12.24	11.59	0.001
ARMD	25	11.60	25.60	20.48	22.88	21.92	15.16	14.22	13.23	0.001
CRVO	18	12.28	31.89	25.67	20.22	19.32	13.75	12.42	11.13	0.001
BRVO	19	11.79	28.16	20.63	23.05	22.32	16.32	13.80	11.46	0.001
UVIETS	5	11.60	33.20	19.60	19.87	18.87	14.87	13.26	10.43	0.001
Vit Hg	19	13.58	36.32	27.05	22.32	21.47	16.54	14.09	13.76	0.001

The mean post-injection IOP at five minutes recorded for DME patients was 27.41 mmHg, which decreased as the time interval changed, and at the sixth hour was found to be 11.24 mmHg. Similarly, we saw variation in mean IOP in PDR patients, as the mean IOP at five minutes was 28.94 mmHg, and at the sixth hour, it reduced to 11.59 mmHg (Table 3).

**Table 3:** Comparison of pre- and post-operative raised IOP ≥ 21 IOP: intraocular pressure p-value was calculated from a comparison of IOP for each post-operative group with pre-operative IOP

IOP	N	IOP mmHg (min)	IOP mmHg (max)	Mean±SD	p-value
Pre Op IOP	191	08	20	12.12±2.11	-
IOP ≥ 21 5 mins later	169	22.00	50.00	30.43±6.53	0.001
IOP ≥ 21 30 mins later	104	22.00	42.00	26.26±4.64	0.001
IOP ≥ 21 1 hr later	33	22.00	36.00	26.12±3.31	0.001
IOP ≥ 21 2 hrs later	16	22.00	30.00	25.62±3.03	0.001

## Discussion

Intravitreal anti-VEGF injections are now an accepted form of treatment for different retinal and choroidal conditions. Many different types of anti-VEGF injections are used for this purpose. Three of them, ranibizumab, aflibercept, and pegaptanib, are the licensed ones, and bevacizumab is used as an off-label drug. Despite being off-label, bevacizumab is commonly used in developing countries due to its cost-effectiveness and easy availability.

As anti-VEGF use has become common, there is always a concern about the adverse effects of the injections on the eye. A study conducted by our group in 2010 by Khan A et al. looked into the ocular complications of Intravitreal bevacizumab injections in eyes with retinal or choroidal neovascularization [13]. Out of these complications, the insignificant elevation of IOP was seen in only two eyes after 12 weeks. Contrasting outcomes are seen in many studies conducted to find out long-term ocular hypertension with Intravitreal bevacizumab. Trabecular alterations after repeated injections, silicone micro droplets, autoimmune reactions, protein aggregation, trabecular congestion, and chronic trabeculations are the mechanisms postulated for the cause of ocular hypertension [14].

The current study looked into the immediate rise in IOP after the first-time Intravitreal injection of bevacizumab. In this study, the IOP at five minutes after the injection was recorded at 30.44 ± 6.53 mmHg (p < 0.001). This was 18 mmHg higher than the baseline level of 12.12 ± 2.11 mmHg. A study conducted to see the immediate post-injection IOP rise by Lemos-Reis et al. in 2014 found a mean rise of 28.6 ± 13.8 mmHg in the absence of sub-conjunctival reflux and only 7.7 ± 10.3 mmHg in the presence of reflux [15]. Our study did not include the analysis of sub-conjunctival reflux of the drug and rather used a cotton applicator to stop the reflux of the drug. This could be the reason for the difference in IOP between the two studies. Kim et al. also investigated the acute rise in the IOP after anti-VEGF injection [16]. His study found an average rise of 30 mmHg, which is also much higher than our study. His study, however, included other drugs like triamcinolone and ranibizumab with different sizes of needles for the injections, and this could be the reason for the difference in our study. Felfeli et al. reported a rise of 26.3 mmHg immediately after the injection [17]. A study by El Chehab et al. reported a mean IOP rise of 46.5 mmHg one minute after the injection [18]. All these studies have varying results as the studies are conducted under different circumstances and with multiple drugs in addition to bevacizumab. Khan et al. in their cohort of 55 patients with DME receiving bevacizumab in a dose of 1.25 mg showed a strong negative linear correlation between the axial length of the eyeball and the extent of raised IOP [19]. The limitation of their findings was that IOPs were recorded one minute before and one minute after the injection using Tono-Pen.

The post-injection IOP spike is generally transient, with pressure returning to normal in a few hours post-injection. In the present study, the IOP returned to less than 21 mmHg three hours after the injection. A meta-analysis of 46 articles by De Varies et al. reported a mean rise of 23.4 mmHg returning to baseline after one week [20]. On the contrary, multiple other studies have shown IOP returning to safer levels within half an hour after the injection without any intervention [21,22]. These varying results can be because of the different types of patient cohorts used in the studies. Our study excluded all the patients with a personal or family history of glaucoma and use of triamcinolone and any anti-VEGF in the past and studied the effects of intravitreal bevacizumab on the IOP. In addition to previous glaucoma and ocular volume, other potential risk factors for a sustained rise in IOP can be multiple injections, the volume of injected medication, intraocular fluid dynamics, and increased outflow facility [23].

In the current study, the mean IOP at five minutes for patients with DME was 27.41 mmHg, which decreased to 11.24 mmHg in the sixth hour after the injection. Similarly, the mean IOP in patients with PDR at five minutes was 28.94 mmHg and decreased to 11.59 mmHg at six hours (Table 5). The most common reasons for receiving intravitreal injections in this study were DME (36.1%) and PDR (18.8%), which is why the rise in IOP was seen more in these conditions. The patient cohort in other studies mentioned in this discussion is mostly ARMD patients, which involves a different patient cohort.

The study being discussed here reported a pressure spike in 88.1% of patients in the first five minutes after injection, which is much higher than the study by Lemos-Reis et al. (32% of patients) and Kim et al. (36% of patients) [15,16]. As mentioned earlier, our study did not include an analysis of subconjunctival reflux of the medication after injection, which could be the cause of the difference in the number of patients showing a pressure spike.

In our study, the average age of patients was 54.5 ± 8.8 years, which is much younger than the mean age of 75 years in the study by Lemos-Reis et al. and the mean age of 69 in a study by Sobaci et al. [15,24]. The comparatively younger age group in our study may be due to the majority of patients having diabetic eye disease rather than ARMD. This needs further investigation in future research to plan for long-term diabetic eye disease control strategies.

Intravitreal anti-VEGF treatment is here to stay until we find other possible routes of drug delivery in the retina. An IOP spike is a reality in the majority of patients after intravitreal drug delivery, and the height of IOP can be alarming, as seen in our study and others. Patients with a history of glaucoma are at real risk of this pressure spike, as sudden increases in IOP can have a deteriorating effect on optic disc function.

One limitation of our study is that we did not consider the axial length of the eyes in which the intravitreal drug was deposited.

## Conclusions

The initial administration of bevacizumab via intravitreal injection may cause a transient elevation in IOP, which typically resolves within a few hours. However, patients with pre-existing glaucoma or those who undergo multiple injections may experience permanent impairments of the retinal nerve fiber layer. In these cases, medical or surgical interventions should be employed to mitigate pressure spikes.
